# Supplementary material for: Non-dinosaurian dinosauromorphs from the Chinle Formation (Upper Triassic) of the Eagle Basin, northern Colorado: Dromomeron romeri (Lagerpetidae) and a new taxon, Kwanasaurus williamparkeri (Silesauridae)
Source: PeerJ. 2019 Sep 3;7:e7551. doi: 10.7717/peerj.7551 (PMC6730537; doi:10.7717/peerj.7551)
Supplement: Supplemental Information 1 [file peerj-07-7551-s001.docx]

**APPENDIX 1: DESCRIPTION OF MEASUREMENTS OF APPENDICULAR ELEMENTS**

See Table S1 for measurements and Figure S1 for graphic representations of measurements.

**Scapula** (Fig. S1H-J; also see Fig. 5)

1. Greatest length, measured from ventral coracoid articulation to dorsal apex
2. Mediolateral width across articular glenoid
3. Anteroposterior length across articular glenoid
4. Anteroposterior thickness at midpoint of shaft
5. Mediolateral width at midpoint of shaft
6. Greatest preserved anteroposterior width of dorsal end
7. Greatest preserved mediolateral width of dorsal end

**Humerus** (Fig. S1D-G, O-R; also see Fig. 15)

1. Proximal width between ectotuberosity and medial tuberosity (only available for *Dromomeron*)
2. Greatest proximal thickness across endotuberosity (only available for *Dromomeron*)
3. Anteroposterior thickness of deltopectoral crest
4. Maximum length from proximal end to distal end
5. Maximum mediolateral width across dorsopectoral crest
6. Mediolateral width at midshaft
7. Anteroposterior width at midshaft
8. Maximum width at distal end
9. Anteroposterior thickness across entepicondyle
10. Anteroposterior thickness across ectepicondyle
11. Anteroposterior thickness of distal end between distal condyles

**Ilium** (Fig. S1W-X; also see Figs. 16-17)

1. Maximum anteroposterior length iliac blade from anterior tip of the preacetabular process to posterior tip of postacetabular process
2. Maximum anteroposterior length of lower ilium between anterior edge of pubic peduncle and posterior edge of ischium peduncle
3. Maximum mediolateral width acetabulum from edge of supracetabular crest to medial edge of ilium
4. Dorsoventral height of ilium between lower edge of supracetabular crest and dorsal edge of iliac blade

**Femur** (Fig. S1A-C, S-V; also see Figs. 18-22)

1. Maximum width of proximal end between posterolateral edge and anteromedial tuber,
2. Maximum thickness across anterolateral tuber
3. Maximum length between proximal and distal ends
4. Maximum mediolateral width across anterior trochanter
5. Anteroposterior width at midshaft
6. Mediolateral width at midshaft
7. Maximum mediolateral width at distal end
8. Maximum distal length across medial condyle
9. Maximum distal length across crista tibiofibularis
10. Maximum distal length of sulcus between condyles

**Tibia** (Fig S1K-N; also see Figs. 6-7)

1. Anteroposterior length at proximal end between cnemial crest and posterior edge
2. Maximum mediolateral width at distal end between proximal condyles
3. Maximum length of tibia between proximal and distal ends
4. Distance from proximal end to distal end of fibular crest
5. Anteroposterior length at midshaft
6. Mediolateral width at midshaft
7. Anteroposterior length at distal end across posterolateral process
8. Mediolateral width of distal end

**APPENDIX 2: ADDITIONAL CODINGS ADDED TO PEECOOK ET AL. (2013) DATA MATRIX**

The matrix of *Peecook et al. (2013)* was used with modifications discussed in the text. Complete codings for *Kwanasaurus*, *Technosaurus*, and *Soumyasaurus* are given below based on the information presented here as well as *Sarigül, Agnolin, & Chatterjee (2018)*. Additional codings for *Dromomeron*, *Eucoelophysis,* and *Lutungutali* are based on new information provided here, our observations of GR 224 and GR 225, and *Peecook et al. (2017)* respectively*.* The character numbers in the *Peecook et al. (2013*) matrix do not all match those provided by *Nesbitt et al. (2010)*. It is assumed that *Peecook et al.’s (2013)* description of the character and its states reflect how it was coded for taxa in their analysis; discrepancies with the numbering of *Nesbitt et al., (2010)* are noted where they occur. Character state 85 was changed to “?” for *Lewisuchus/Pseudolagosuchus*, for which the dentary is probably unknown (*Bittencourt et al., 2015*). Unknown character states are not discussed.

**CHARACTERS ADDED TO THE PEECOOK ET AL. (2013) MATRIX.**

- **292 (formerly 291 from *Kammerer, Nesbitt & Shubin, 2012*)**. Dentition, anterior portion of the dentary, teeth remain relatively same size throughout anterior portion of dentition (0); teeth significantly decrease in size anteriorly (1).
- **293 (formerly 292 from *Kammerer, Nesbitt & Shubin, 2012*)**. Dentition, anterior portion of the dentary, long axis of the teeth, vertical (0); inclined anteriorly (1).
- **294 (new character).** Lateral surface of dentary, lacks longitudinal ridge (0); possesses a longitudinal ridge (1).

Note: Characters 292 and 293were coded as “1” only for *Sacisaurus* and *Diodorus* in the original analysis of *Kammerer, Nesbitt & Shubin (2012)*. Although an anterior decrease in tooth size is also detectable in *Technosaurus*, *Silesaurus*, and *Kwanasaurus,* the extreme decrease in size seen in *Diodorus* and *Sacisaurus* can only be documented in *Kwanasaurus*. The decrease in size is not as extreme in *Silesaurus*, which is presumably why *Kammerer, Nesbitt & Shubin (2012)* coded it as “0”. Moreover, it is unclear if the anteriormost dentary teeth were as small in *Technosaurus* as the anteriormost dentary teeth of the other taxa coded with the derived state, and for *Technosaurus* the character is therefore coded as “?” as in other silsaurids, in which the anterior tip of the dentary or the entire dentary is missing (Fig. 10). Likewise canting (anterior orientation) of the anteriormost teeth is detectable in *Kwanasaurus* as well as *Diodorus* and *Sacisaurus*, but not known in other silesaurid taxa (Fig. 10); of these, only *Silesaurus* can be definitively coded as “0” (absence of canting), with all other taxa being coded as “?” due to the anterior dentary teeth being missing. In the matrix, the plesiomorphic state was coded for both characters for all other taxa possessing anterior dentary teeth, following *Kammerer, Nesbitt & Shubin (2012).* Character 294 can be coded for more taxa, as the dentary is one of the best-represented elements within Silesauridae, with the derived state occurring only in *Diodorus*, *Eucoelophysis*, and *Kwanasaurus*.

**COMPLETE CODINGS**

***Kwanasaurus williamparkeri***

- 11(0): Maxilla, facial portion anterior to anterior edge of antorbital fenestra shorter than posterior portion (character 9 in Nesbitt et al., 2010).
- 12(1): Maxillary teeth, posterior edge of posterior maxillary teeth concave or straight (character 10 in Nesbitt et al., 2010).
- 13(0): Maxilla, posterior process articulates ventral to jugal (character 11 in Nesbitt et al., 2010).
- 14(0): Maxilla, interdental plates separate (character 12 in Nesbitt et al., 2010).
- 15(0): Maxilla, absence of buccal emargination separated from the ventral emargination of the antorbital fossa (character 13 in Nesbitt et al., 2010).
- 16(0): Maxilla, anterodorsal margin at the base of the dorsal process convex or straight (character 15 in Nesbitt et al., 2010).
- 18(0): Maxilla, lateral surface smooth (character 16 in Nesbitt et al., 2010).
- 19(1): Maxilla, posterior portion ventral to antorbital fenestra has similar dorsoventral depth to the anterior portion ventral to the antorbital fenestra (character 17 in Nesbitt et al., 2010).
- 20(0): Maxilla, promaxillary foramen absent (character 18 in Nesbitt et al., 2010).
- 22(0): Maxilla, anterior margin of antorbital fenestra gently rounded (character 20 of Nesbitt et al., 2010).
- 23(1): Maxilla, palatal processes meet at the midline (character 21 of Nesbitt et al., 2010). Note: This is not completely certain given that there are no complete skulls known for *Kwanasaurus*, but seems likely given the size of the medial flange.
- 78(2): Maxilla, antorbital fossa present on the lacrimal, dorsal process of the maxilla, and the dorsolateral margin of the posterior process of the maxilla. Note: The lacrimal is unknown for *Kwanasaurus*, but the different character states deal with the participation of the maxilla.
- 85(1): Dentary, Meckelian groove restricted to ventral border of dentary.
- 86(0): Dentary, Meckelian groove does not extend through dentary symphysis.
- 87(2): Dentary, dorsal margin of anterior portion dorsally expanded compared to the posterior part of the dentary. Note: This expansion is slight in *Kwanasaurus*, but no less so than in *Silesaurus*, which Nesbitt et al. (2010) assigned the same character state.
- 88(2): Dentary, anterior extremity tapers to a sharp point.
- 96(1): Dentary, anterior dentary teeth absent.
- 97(0): Dentition, generally homodont.
- 98(2): Dentition, tooth serrations are enlarged and coarsened denticles.
- 99(0): Dentition, Absence of extensive planar wear facets across multiple maxillary and/or dentary teeth.
- 100(0 & 1): Dentition, medial or lateral overlap of adjacent crowns in maxilla and dentary. Note: Overlap has not been documented in dentary teeth, but does occur in the maxillary teeth.
- 101(1): Dentition, crowns mesiodistally expanded above root in cheek teeth.
- 102(1): Dentition, moderately developed lingual expansion of crown (cingulum) on maxillary/dentary teeth. Note: The lingual expansion is quite moderate compared to *Pisanosaurus* and ornithischians, but similar to the condition in *Sacisaurus*, which is coded with the derived state.
- 103(1): Dentition, tooth crowns apicobasally short and subtriangular.
- 104(0): Dentition, teeth fused to socket. Note: The coding is consistent with Nesbitt et al. (2010) but the opposite of Nesbitt (2011, character 174), where fused teeth are coded as derived instead of plesiomorphic.
- 126(0): Sacral vertebrae, rib of first primordial sacral does not articulate with preacetabular process.
- 138(1): Scapula, entire anterior margin markedly concave.
- 139(1): Scapula, blade height more than three times distal width.
- 144(1): Scapula, glenoid directed posteroventrally.
- 145(0): Humerus, apex of deltapectoral crest less than 30% of the length of the humerus from the proximal end.
- 147(0): Humerus, proximal articular surface isn’t hooked and doesn’t extend onto posterior surface.
- 148(0): Humerus, proximal articular surface continuous with deltopectoral crest.
- 149(1): Humerus, no ectepicondylar flange.
- 150(0): Humerus, distal end narrow or equal to 30% of length of humerus.
- 173(0): Ilium, supraacetabular crest projects laterally or ventrolaterally.
- 174(0): Ilium, no crest dorsal to the supra-acetabular crest ,
- 175(1): Ilium, preacetabular process long and extends anterior to the acetabulum.
- 176(0): Ilium, mainly vertically oriented (0**°**-20**°**).
- 177(1): Ilium, distinct fossa for the attachment of the M. caudifemoralis brevis present as an embankment on the lateral side of the posterior portion of the ilium.
- 178(0): Ilium, ridge connecting the posterior portion of the supra-acetabular rim to the posterior portion of the illium.
- 179(2): Illium, ventral margin of the acetabulum concave.
- 180(0): Illium, acetabular antitrochanter absent.
- 181(0): Ilium, dorsal margin of supraacetabular rim rounded or blade-like.
- 182(0): Ilium, height of dorsal portion about the same or shorter than the distance between the supra-acetabular rim to the pubis-ischium contact.
- 183(0): Ilium, ischiadic peduncle mainly with a vertical orientation in lateral view.
- 203(1): Femur, anteromedial tuber on proximal portion small and rounded.
- 204(2): Femur, posteromedial tuber absent on the proximal portion.
- 205(0): Femur, anterolateral tuber present as an expansion on the proximal portion.
- 206(1): Femur, medial articular surface of the proximal end is flattened.
- 207(1): Femur, notch present ventral to the proximal head.
- 208(1): Femur, head oriented anteromedially at about 20**°**-60°.
- 209(0): Femur, head is rounded in medial and lateral views.
- 210(1): Femur, dorsolateral margin of the proximal portion is a sharp ridge (the dorsolateral trochanter).
- 211(1): Femur, anterior trochanter (insertion for the M. iliofemoralis cranialis) present and forms a steep margin with the shaft although it is completely connected to it. Note: This is the only character state for the anterior trochanter that is unambiguously present in at least some specimens of *Kwanasaurus*, although it is possible that character state 2 (the anterior trochanter is notched) occurs in some specimens; however, this cannot yet be confirmed.
- 212(1): Femur, medial articular facet on proximal end is straight.
- 213(0): Femur, anterolateral side of the femoral head is smooth and featureless.
- 214(0): Femur, absence of an anterior trochanteric shelf proximal to the fourth trochanter.
- 215(0): Femur, posterolateral portion (fossa trochanteria, posterolateral depression, facies antitrochanterica articularis) of head level with greater trochanter.
- 216(1): Femur, proximal surface with straight, transverse groove.
- 217(0): Femur, fourth trochanter mound-like and rounded.
- 218(0): Femur, fourth trochanter symmetrical, with distal and proximal margins forming similar low angle slopes to the shaft.
- 219(0): Femur, angle between the lateral condyle and crista tibiofibularis is obtuse in distal view.
- 220(0): Femur, distal medial condyle tapers to a point and is medially oriented. Note: The medial condyle is very sharp, making it unique among silesaurids; therefore, even though it is more laterally oriented than medially oriented, it is coded as the plesiomorphic state.
- 221(1): Femur, distal surface between the lateral condyle and crista tibiofibularis is a deep groove.
- 223(1): Femur, distal condyles divided posteriorly between 1/4 and 1/3 the length of the shaft.
- 224(0): Femur, anterior surface of the distal portion is smooth.
- 225(1): Femur, crista tibiofibularis (fibular condyle) is larger than the medial condyle.
- 226(0): Femur, anterolateral corner of distal end is round.
- 227(1): Tibia, cnemial crest on the proximal end is present and straight.
- 228(0): Tibia, proximal surface flat or concave.
- 229(0): Tibia, proximal surface of lateral condyle convex or flat.
- 230(1): Tibia, lateral (fibular) condyle level with the medial condyle on the posterior surface of the proximal end.
- 231(0): Tibia, lateral margin of the lateral condyle on the proximal end is rounded.
- 232(1): Tibia, lateral side of proximal portion bears a dorsoventrally oriented fibular crest.
- 233(1): Tibia, posterolateral flange of distal end is present and extends will posterior to the fibula.
- 234(0): Tibia, posterolateral portion of the distal end is straight or convex.
- 235(0): Tibia, posterior face of the distal end is a rounded surface.
- 236(0): Tibia, posterior side of the distal portion smooth and featureless.
- 237(0): Tibia, lateral side of distal end is smooth or rounded.
- 292 (1): Dentition, anterior portion of the dentary, teeth significantly decrease in size anteriorly.
- 293 (1): Dentition, anterior portion of the dentary, long axis of the teeth inclined anteriorly.
- 294 (1): Longitudinal ridge on lateral surface of dentary present.

***Diodorus scytobrachion*** (from *Kammerer, Nesbitt, & Shubin, 2012*)

- 85(1): Dentary, Meckelian groove restricted to ventral border of dentary.
- 86(0): Dentary, Meckelian groove does not extend through dentary symphysis.
- 97(0): Dentition, generally homodont.
- 98(2): Dentition, tooth serrations are enlarged and coarsened denticles.
- 99(0): Dentition, Absence of extensive planar wear facets across multiple maxillary and/or dentary teeth.
- 101(1): Dentition, crowns mesiodistally expanded above root in cheek teeth.
- 102(0): Dentition, no lingual expansion of crown (cingulum) on maxillary/dentary teeth.
- 103(1): Dentition, tooth crowns apicobasally short and subtriangular.
- 104(0): Dentition, teeth fused to socket. Note: The coding is consistent with Nesbitt et al. (2010) but the opposite of Nesbitt (2011, character 174), where fused teeth are coded as derived instead of plesiomorphic.
- 145(0): Humerus, apex of deltapectoral crest less than 30% of the length of the humerus from the proximal end.
- 147(0): Humerus, proximal articular surface isn’t hooked and doesn’t extend onto posterior surface.
- 148(0): Humerus, proximal articular surface continuous with deltopectoral crest.
- 149(1): Humerus, no ectepicondylar flange.
- 150(0): Humerus, distal end narrow or equal to 30% of length of humerus.
- 203(1): Femur, anteromedial tuber on proximal portion small and rounded.
- 204(2): Femur, posteromedial tuber absent on the proximal portion.
- 205(1): Femur, anterolateral tuber present as an expansion on the proximal portion.
- 206(1): Femur, medial articular surface of the proximal end is flattened.
- 207(1): Femur, notch present ventral to the proximal head.
- 209(0): Femur, head is rounded in medial and lateral views.
- 210(1): Femur, dorsolateral margin of the proximal portion is a sharp ridge (the dorsolateral trochanter).
- 212(1): Femur, medial articular facet on proximal end is straight.
- 213(0): Femur, anterolateral side of the femoral head is smooth and featureless.
- 215(0): Femur, posterolateral portion (fossa trochanteria, posterolateral depression, facies antitrochanterica articularis) of head level with greater trochanter.
- 216(0): Femur, proximal surface rounded and smooth.
- 217(1): Femur, fourth trochanter a sharp flange.
- 218(0): Femur, fourth trochanter symmetrical, with distal and proximal margins forming similar low angle slopes to the shaft.
- 219(0): Femur, angle between the lateral condyle and crista tibiofibularis is obtuse in distal view.
- 220(1): Femur, distal medial condyle smoothly rounded in distal view.
- 221(0): Femur, distal surface between the lateral condyle and crista tibiofibularis is smooth.
- 223(1): Femur, distal condyles divided posteriorly between 1/4 and 1/3 the length of the shaft.
- 224(0): Femur, anterior surface of the distal portion is smooth.
- 225(0): Femur, crista tibiofibularis (fibular condyle) is smaller or of equal size to medial condyle.
- 226(0): Femur, anterolateral corner of distal end is rounded.
- 292 (1): Dentition, anterior portion of the dentary, teeth significantly decrease in size anteriorly.
- 293 (1): Dentition, anterior portion of the dentary, long axis of the teeth inclined anteriorly.
- 294 (1): Longitudinal ridge on lateral surface of dentary present.

***Ignotosaurus fragilis*** (from *Martinez et al., 2012*)

- 173(0): Ilium, supraacetabular crest projects laterally or ventrolaterally.
- 174(2): Ilium, crest dorsal to supraacetabular crest confluent with anterior extent of preacetabular process.
- 175(0): Ilium, preacetabular process short and does not extend anterior to the acetabulum.
- 176(0): Ilium, mainly vertically oriented (0**°**-20**°**).
- 177(2): Ilium, distinct fossa for the attachment of the M. caudifemoralis brevis present as a deep fossa on the ventral surface of the postacetabular part of the ilium.
- 178(0): Ilium, ridge connecting the posterior portion of the supra-acetabular rim to the posterior portion of the illium.
- 179(1): Illium, ventral margin of the acetabulum straight.
- 180(0): Illium, acetabular antitrochanter absent.
- 181(0): Ilium, dorsal margin of supraacetabular rim rounded or blade-like.
- 182(1): Ilium, height of dorsal portion expanded taller than than the distance between the supra-acetabular rim to the pubis-ischium contact.
- 183(0): Ilium, ischiadic peduncle mainly with a vertical orientation in lateral view.
- 184(1): Pubis length more than 70% of femoral length.

***Technosaurus smalli***

- 85(1): Dentary, Meckelian groove restricted to ventral border of dentary.
- 97(0): Dentition, generally homodont. Note: The tooth crowns are not well-preserved, but seem to be of roughly the same form.
- 98(2): Dentition, tooth serrations are enlarged and coarsened denticles. Note: The teeth are poorly preserved in *Technosaurus*, but denticles can be discerned on some of the crowns.
- 100(0 and1): Dentition, medial or lateral overlap of adjacent crowns in maxilla and dentary. Note: Overlap occurs in the anteriormost dentary teeth.
- 101(1): Dentition, crowns mesiodistally expanded above root in cheek teeth.
- 102(1): Dentition, moderately developed lingual expansion of crown (cingulum) on maxillary/dentary teeth. Note: The lingual expansion is quite moderate compared to *Pisanosaurus* and ornithischians, but similar to the condition in *Sacisaurus*, which is coded with the derived state; contrary to *Martz et al. (2013)* we therefore identify it as present.
- 103(1): Dentition, tooth crowns apicobasally short and subtriangular.
- 104(0): Dentition, teeth fused to socket. Note: The coding is consistent with Nesbitt et al. (2010) but the opposite of Nesbitt (2011, character 174), where fused teeth are coded as derived instead of plesiomorphic.
- 294 (0): Longitudinal ridge on lateral surface of dentary absent.

***Soumyasaurus aenigmaticus***

- 85(1): Dentary, Meckelian groove restricted to ventral border of dentary.
- 86(0): Dentary, Meckelian groove does not extend through dentary symphysis. Note: although the anterior tip of the dentary is missing, the Meckelian groove seems to become extremely small and shallow near the preserved end.
- 100(0): Dentition, medial or lateral overlap of adjacent crowns in maxilla and dentary absent. Note: Few teeth are preserved, but their size, shape, and spacing makes any overlap unlikely.
- 101(0): Dentition, crowns not mesiodistally expanded.
- 102(0): Dentition, moderately developed lingual expansion of crown (cingulum) on maxillary/dentary teeth. Note: The small, simple teeth of *Soumyasaurus* do not seem to possess any lingual expansion.
- 103(1): Dentition, tooth crowns apicobasally short and subtriangular.
- 104(0): Dentition, teeth fused to socket. Note: The coding is consistent with Nesbitt et al. (2010) but the opposite of Nesbitt (2011, character 174), where fused teeth are coded as derived instead of plesiomorphic.
- 294 (0): Longitudinal ridge on lateral surface of dentary absent.

**ADDITIONAL CODINGS**

***Dromomeron romeri***

- 145(0): Humerus, deltopectoral crest less than 30% down the length of the humerus.
- 147(0): Humerus, proximal head confined to proximal surface.
- 148(1): Humerus, proximal articular surface and deltopectoral crest separated by a thin crest.
- 149(0): Humerus, ectepicondylar flange absent.
- 150(0): Humerus, distal end less than or equal to 30% humerus length.

***Eucoelophysis baldwini***

- 85(1): Dentary, Meckelian groove restricted to ventral border of dentary.
- 86(0): Dentary, Meckelian groove does not extend through dentary symphysis.
- 98(2): Dentition, tooth serrations are enlarged and coarsened denticles.
- 99(0): Dentition, Absence of extensive planar wear facets across multiple maxillary and/or dentary teeth.
- 101(1): Dentition, crowns mesiodistally expanded above root in cheek teeth.
- 102(1): Dentition, moderately developed lingual expansion of crown (cingulum) on maxillary/dentary teeth. Note: The lingual expansion is quite moderate compared to *Pisanosaurus* and ornithischians, but similar to the condition in *Sacisaurus*, which is coded with the derived state.
- 103(1): Dentition, tooth crowns apicobasally short and subtriangular.
- 104(0): Dentition, teeth fused to socket. Note: The coding is consistent with Nesbitt et al. (2010) but the opposite of Nesbitt (2011, character 174), where fused teeth are coded as derived instead of plesiomorphic.
- 173(0): Ilium, supraacetabular crest projects laterally or ventrolaterally.
- 174(0): Ilium, no crest dorsal to the supra-acetabular crest ,
- 175(0): Ilium, preacetabular process short and does not extend anterior to the acetabulum.
- 176(0): Ilium, mainly vertically oriented (0**°**-20**°**).
- 177(1): Ilium, distinct fossa for the attachment of the M. caudifemoralis brevis present as an embankment on the lateral side of the posterior portion of the ilium.
- 178(0): Ilium, ridge connecting the posterior portion of the supra-acetabular rim to the posterior portion of the Ilium.
- 181(0): Ilium, dorsal margin of supraacetabular rim rounded or blade-like.
- 182(0): Ilium, height of dorsal portion about the same or shorter than the distance between the supra-acetabular rim to the pubis-ischium contact.
- 294 (1): Longitudinal ridge on lateral surface of dentary present.

***Lutungutali sitwensis*** (from *Peecook et al., 2017*)

- 14(0): Maxilla, interdental plates separate (character 12 in Nesbitt et al., 2010).
- 15(0): Maxilla, absence of buccal emargination separated from the ventral emargination of the antorbital fossa (character 13 in Nesbitt et al., 2010).
- 20(0): Maxilla, promaxillary foramen absent (character 18 in Nesbitt et al., 2010).
- 22(0): Maxilla, anterior margin of antorbital fenestra gently rounded (character 20 of Nesbitt et al., 2010).
- 104(0): Dentition, teeth fused to socket. Note: The coding is consistent with Nesbitt et al. (2010) but the opposite of Nesbitt (2011, character 174), where fused teeth are coded as derived instead of plesiomorphic.
- 203(1): Femur, anteromedial tuber on proximal portion small and rounded.
- 204(2): Femur, posteromedial tuber absent on the proximal portion.
- 205(0): Femur, anterolateral tuber present as an expansion on the proximal portion.
- 206(1): Femur, medial articular surface of the proximal end is flattened.
- 207(1): Femur, notch present ventral to the proximal head.
- 208(1): Femur, head oriented anteromedially at about 20**°**-60°.
- 209(0): Femur, head is rounded in medial and lateral views.
- 211(1): Femur, anterior trochanter (insertion for the M. iliofemoralis cranialis) present and forms a steep margin with the shaft although it is completely connected to it. Note: This coding is based on *Peecook et al.’s (2017)* description of the trochanter as “mound-like.”
- 212(1): Femur, medial articular facet on proximal end is straight.
- 213(0): Femur, anterolateral side of the femoral head is smooth and featureless.
- 214(0): Femur, absence of an anterior trochanteric shelf proximal to the fourth trochanter.
- 215(0): Femur, posterolateral portion (fossa trochanteria, posterolateral depression, facies antitrochanterica articularis) of head level with greater trochanter.
- 216(1): Femur, proximal surface with straight, transverse groove.
- 217(1): Femur, fourth trochanter a sharp flange. Note: *Peecook et al. (2017)* describe the trochanter as “low and blade-like.”
- 218(0): Femur, fourth trochanter symmetrical, with distal and proximal margins forming similar low angle slopes to the shaft.
- 223(1): Femur, distal condyles divided posteriorly between 1/4 and 1/3 the length of the shaft. Note: Although distal femora were identified by *Peecook et al. (2017)*, this is the only character state specified.
- 249(0): Astragalus, dorsally expanded process on the posterolateral portion of the tibial facet: absent or poorly expanded.
- 250(1): Astragalus, anterior ascending flange (anterior process) present and less than the height of the dorsoventral height of the posterior side of the astragalus.
- 251(1): Astragalus, anterior hollow reduced to a foramen or absent.
- 252(1): Articular facet for the astragalus of the calcaneum lies partially ventral to the fibular facet.
- 253(0): Astragalus, proximal surface lacks a marked rimmed and elliptical fossa posterior to the anterior ascending process.
- 254(1): Astragalus, anteromedial corner shape is acute.
- 255(1): Astragalus, proximal articular facet for fibula occupies less than 0.3 of the transverse width.
- 256(0): Astragalus, posterior groove present.
- 257(0) Astragalus, tibial facet concave or flat.
- 258(1): Astragalus-calcaneum, ventral articular surface concavoconvex with concavity on calcaneum.
- 259(0): Astragalus-calcaneum, articulation free.
- 261(0): Calcaneum, articular facets for the fibula and astragalus connected by a continuous surface.
- 262(0): Calcaneum, calcaneal tuber absent.
- 266(0): Calcaneum, articular surface for the fibula convex.
- 267(0): Calcaneum, shape: proximodistally compressed with a short posterior projection and medial process.
- 268(1): Calcaneum, articular surfaces for fibula and distal tarsal IV continuous.

**APPENDIX 3: CLADE DIAGNOSES**

For the analysis excluding *Ignotosaurus*, *Soumyasaurus*, and *Technosaurus*, PAUP lists the following character state transformations occurring at the following nodes preserved in the strict consensus and Adams consensus tree:

**Silesauridae**

- 6(1🡪2): Five premaxillary teeth (character 5 in Nesbitt et al., 2010).
- 37(0🡪1): Squamosal ventral process narrower than one quarter if its length (character 36 in Nesbitt et al. 2010).
- 66(0🡪1): Exoccipital relative positions of exits of hypoglossal aligned. subvertically (character 65 in Nesbitt et al., 2010).
- 72(0🡪1): Supraoccipital, rugose ridge on anterolateral edges present.
- 104(1🡪0): Tooth implantation, teeth fused to the bone of attachment at base.
- 109(0🡪1): Cervical vertebrae, 3-5 centrum length longer than mid-dorsal.
- 148(1🡪0): Humerus, proximal articular surface continuous with deltopectoral crest.
- 180(1🡪0): Ilium, acetabular antitrochanter absent.
- 216(0🡪1): Femur, proximal surface transverse groove that is straight.
- 282(0🡪1): Pedal ungals, laterally compressed.

***Asilisaurus* + Sulcimentisauria**

- 47(0🡪2): Jugal, longitudinal ridge on the body rounded and broad (character 46 in Nesbitt et al., 2010).
- 56(2🡪0): Parabasisphenoid, foramina for entrance of cerebral branches of internal carotid artery into braincase (character 55 in Nesbitt et al., 2010).
- 88(0🡪1): Dentary, anterior extremity tapers to a sharp point.
- 96(0🡪1): Dentary, teeth absent in anterior portion.
- 103(0🡪1): Maxillary and dentary crowns, shape apibasically short and subtriangular.
- 131(0🡪1): Sacral ribs, shared between two sacral vertebrae.
- 202(1🡪0): Tibia or fibula, femur longer or about the same length.
- 212(0🡪1): Femur, medial articular facet straight.
- 223(0🡪1): Femur, distal condyles divided posteriorly between 1/4 and 1/3 the length of the shaft

**Sulcimentisauria**

- 85 (0🡪1): Meckelian groove restricted to the ventral border of the dentary.
- 87 (1🡪2): Dorsal margin of the anterior portion of the dentary dorsally expanded compared to the dorsal margin of the posterior dentary.
- 111 (1🡪0): Third cervical vertebra centrum length subequal to axis centrum.
- 130 (0🡪1): Insertion of a sacral vertebra between first and second primordial vertebrae.
- 177 (0🡪1): Brevis fossa occurs on ilium as an embankment on the lateral side of the postacetabular process.
- 204 (0🡪2): Posteromedial tuber of absent on proximal end of femur.
- 206 (0🡪1): Medial articular surface of proximal femur is flattened.
- 214 (1🡪0): Anterior trochanter present on femur.
- 215 (1🡪0): Fossa trochanterica of femur level with greater trochanter.
- 230 (0🡪1): Fibular condyle of tibia level with medial condyle at its posterior border.
- 232 (0🡪1): Fibular crest present on lateral side of proximal end of tibia.
- 291 (1🡪2): Cross section shape of ischium shaft thin so that shaft is blade-like.

**Unnamed clade (*Sacisaurus* + *Lutungutali* + *Diodorus* +*Eucoelophysis* + *Kwanasaurus*)**

**Note: Among most parsimonious trees, synapomorphies vary depending on the placement of *Sacisaurus*; those not occurring in all most parsimonious trees are labeled as ambiguous.**

- 98 (1🡪2): Tooth serrations are coarsened denticles
- 102 (0🡪1) (ambiguous): Moderate development of a cingulum
- 252 (0🡪1): Calcaneum, facet for astragalus completely medial to fibular facet
- 255 (0🡪1): Astragalus, proximal articular facet for fibula occupies less than 0.3 of transverse width.
- 292 (0🡪1): Tooth size strongly reduced in anterior dentary.
- 293 (0🡪1): Anteriormost dentary teeth canted.
- 294 (0🡪1) (ambiguous): Longitudial ridge on lateral surface of dentary.

**Unnamed African clade (*Lutungutali* + *Diodorus*)**

**Note: Among most parsimonious trees, synapomorphies vary depending on the placement of *Sacisaurus*; those not occurring in all most parsimonious trees are labeled as ambiguous.**

- 102 (1🡪0) (ambiguous): Loss of cingulum on maxillary and dentary teeth
- 182 (0🡪1): Dorsal portion of ilium expanded and markedly taller that the dorsal portion of the supracetabular rim to the pubis-ischium contact.
- 217 (0🡪1): Fourth trochanter of femur a sharp flange.

**Unnamed North American clade (*Eucoelophysis* + *Kwanasaurus*)**

**Note: Among most parsimonious trees, synapomorphies vary depending on the placement of *Sacisaurus*; those not occurring in all most parsimonious trees are labeled as ambiguous.**

- 19 (0🡪1) (ambiguous): Ascending process of the maxilla remains the same width.
- 88 (1🡪2) (ambiguous): Anterior extremity of dentary tapers to a sharp point.
- 174 (2🡪0): Crest of ilium dorsal to the supra-acetabular crest absent.
- 220 (1🡪0) (ambiguous): Medial distal condyle of the femur tapers to a point.
- 221 (0🡪1) (ambiguous): Deep groove between distal condyles on femur.
- 237 (1🡪0) (ambiguous): Lateral side of distal end of femur smooth/rounded.
