# Supplementary figures and images for: Non-dinosaurian dinosauromorphs from the Chinle Formation (Upper Triassic) of the Eagle Basin, northern Colorado: Dromomeron romeri (Lagerpetidae) and a new taxon, Kwanasaurus williamparkeri (Silesauridae)
Source: PeerJ. 2019 Sep 3;7:e7551. doi: 10.7717/peerj.7551 (PMC6730537; doi:10.7717/peerj.7551)

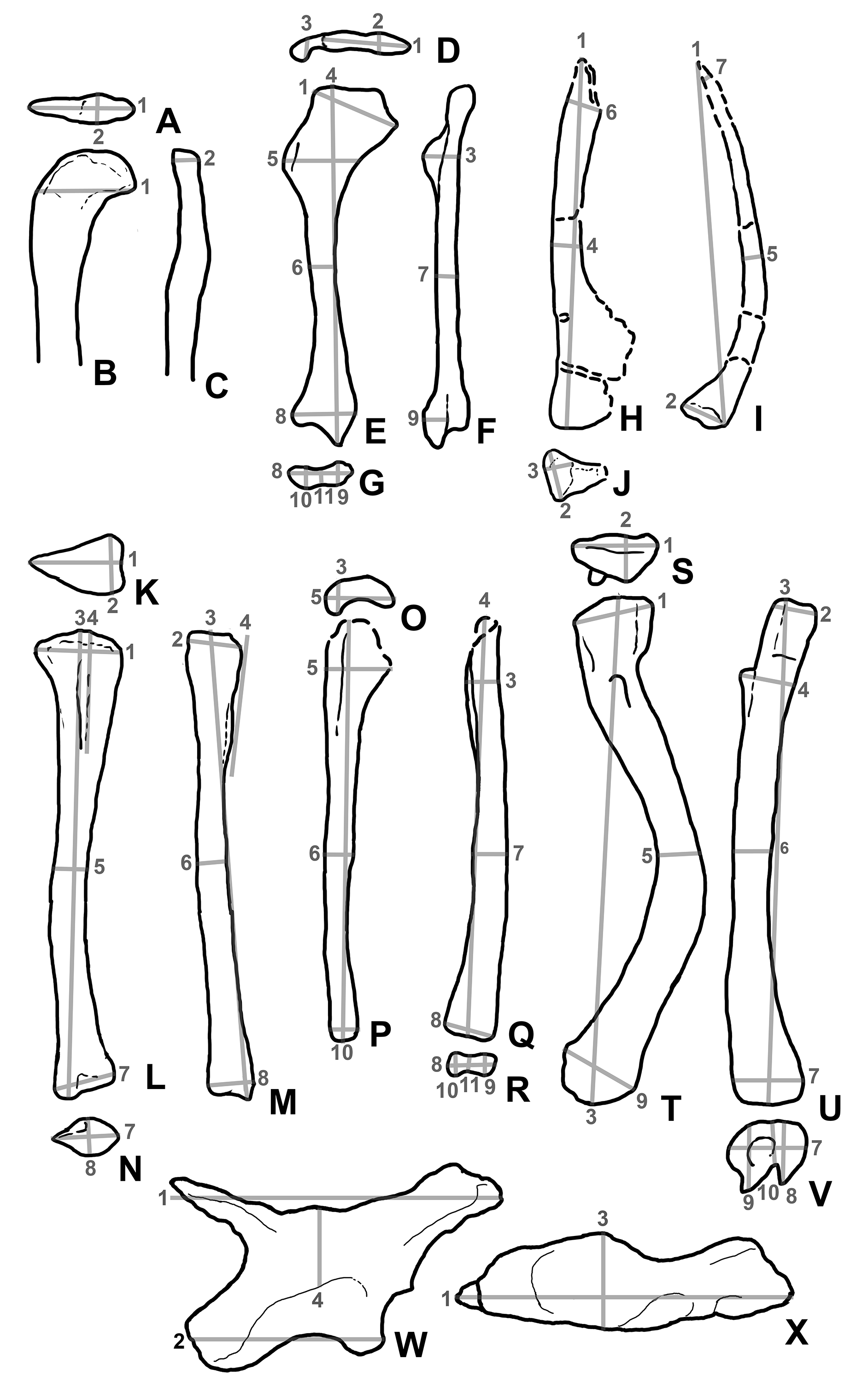

Supplement: Supplemental Information 2 — (A) Dromomeron romeri proximal femur in proximal view, (B) posteromedial view, (C) posterolateral view. (D) Dromomeron romeri humerus in proximal view, (E) anterior view, (F) medial view, (G) distal view. (H) Dinosauriformes scapula in lateral view, (I) posterior view, (J) ventral view. (K) Dinosauriformes tibia in proximal view, (L) lateral view, (M) posterior view, (N) distal view. (O) Silesauridae humerus in proximal view, (P) anterior view, (Q) medial view, (R) distal view. (S) Silesauridae femur in proximal view, (T) anteromedial view, (U) anteromedial view, (V) distal view. [file peerj-07-7551-s002.png]
